# Obesity and metabolic syndrome in 7-9 years-old Portuguese schoolchildren

**DOI:** 10.1186/1758-5996-2-40

**Published:** 2010-06-10

**Authors:** Carla Pedrosa, Bruno MPM Oliveira, Isabel Albuquerque, Carlos Simões-Pereira, Maria D Vaz-de-Almeida, Flora Correia

**Affiliations:** 1 Faculty of Nutrition and Food Sciences of University of Porto. Rua Dr. Roberto Frias, 4200-465 Porto, Portugal; 2 Department of Endocrinology, Diabetes and Nutrition; Hospital Infante D. Pedro, EPE. Avenida Artur Ravara, 3814-501 Aveiro, Portugal; 3 Department of Endocrinology; Hospital de S. João. Alameda Prof. Hernâni Monteiro, 4200-319 Porto, Portugal

## Abstract

**Background:**

Body fat is related to changes in lipid profile, blood pressure and metabolism of insulin and glucose, known as the metabolic syndrome (MS). The aim of this study was to estimate the prevalence of metabolic syndrome (MS) and its components among overweight and obese Portuguese schoolchildren, and to identify associated clinical and biochemical characteristics.

**Methods:**

A total of 82 children (14 overweight and 68 obese; 40 boys and 42 girls) aged 7-9 years, underwent anthropometric measurements. A blood sample was obtained to assess biochemical parameters. Insulin resistance (IR) was determined by the Homeostasis Model Assessment of Insulin Resistance (HOMA-IR). MS was defined by the National Cholesterol Education Program Adult Treatment Panel III criteria modified by Cook.

**Results:**

The prevalence of MS was 15.8%. Abdominal obesity was present in all children. Frequency of elevated blood pressure, low HDL-cholesterol and elevated triglyceride concentrations were 62.6%, 13.4% and 11.0%, respectively. None of the children presented impaired fasting glucose, however hyperinsulinemia (7.3%) and IR (8.5%) were observed. The number of components of MS was higher in children with higher z-BMI (ρ = 0.411; p < 0.001). MS was associated with higher leptin concentrations. No association was found with adiponectin or ghrelin levels. Leptin correlated positively with obesity, glucose metabolism, lipid profile, hepatic function and C-reactive protein, and negatively with HDL and Apolipoprotein A-I/B ratio.

**Conclusions:**

This study shows a significant prevalence of MS among obese schoolchildren. Abdominal obesity and elevated blood pressure were the most frequent components of this syndrome. Dyslipidemia, IR and high levels of leptin were also associated with MS in this young group.

## Background

In the past decades the prevalence of childhood obesity has increased worldwide, reaching epidemic proportions and becoming a serious and growing public health problem [1,2]. In Portugal, in a nationwide study, the prevalence of excess weight in 7-9 year-old children was 31.5% [3]. This is similar to what we found in a previous local study of 920 children from Aveiro (28%) [4].

The coexistence of obesity and metabolic disorders such as hyperinsulinemia, glucose intolerance, hypertension, high serum levels of triglycerides (TG) and decreased levels of high density lipoprotein (HDL), is known as metabolic syndrome (MS) [5]. This constellation of symptoms, first described by Reaven [6], increases the risk for cardiovascular disease (CVD) and type 2 diabetes (DM2) [7]. Insulin resistance (IR) and an altered plasma lipid pattern are common pathophysiological features of MS, not only in adults but also in children and adolescents [7]. However, there is no consensus about the definition of MS in paediatric populations [8]. Many different MS criteria have been employed in children and adolescents, and the components and cut-offs used to diagnose this syndrome have varied considerably among studies [8-10]. Recently, a new consensus definition has been published, however according to it, in children younger than 10 years-old MS should not be diagnosed [11]. Changes in growth and development during childhood and adolescence make it difficult to choose a cut-off for risk factors [12]. Therefore, the prevalence varies according to the definitions used [8]. It has been reported to be relatively low in normal weight children (1% or less) contrary to what occurs in obese (18% to 50%) [13]. Moreover, MS is rapidly increasing in prevalence with rising childhood obesity and sedentary lifestyles worldwide [13]. In addition, parameters associated with MS have been shown to originate early in life [9], and tend to track into adulthood [14]. The recognition of MS in obese children, who have not yet developed cardiometabolic disorders, is of great importance from a clinical and public health perspective [15].

The MS has been widely studied in adults, however there is little research focusing on younger children (< 10 y old) [16]. Moreover, the absence of studies on MS in the paediatric Portuguese population led us to design the current study. The aims of this study were to determine the prevalence of MS and its individual components in a sample of Portuguese obese schoolchildren, and to identify clinical or biochemical characteristics associated with MS in our population.

## Methods

### Study Population

Recruitment of the study sample was population-based [4]. In brief, from a total of forty schools with 1799 schoolchildren enrolled at 2^nd ^and 3^rd ^grade (aged between 7-9 years), a random sample of 13 schools was selected and a representative sample of 980 children was obtained. Weight, height and waist circumference were evaluated in 905 children (457 boys and 448 girls). Of those, 127 (14%) were classified as obese (≥ 95^th ^BMI percentile) according to the US Centers for Disease Control and Prevention (CDC) criteria [17], and were invited to participate in the study. Eighty-nine accepted the invitation and 82 (64.5% of the initial sample; 40 boys and 42 girls) allowed collection of blood.

Each parent gave written informed consent and children gave assent for participation. The study was approved by the Ethics Committee of the Hospital Infante D. Pedro. Assessments were carried out at the Department of Endocrinology, Diabetes and Nutrition.

None of the evaluated children suffered from primary dyslipidemia, hypertension, diabetes or glucose intolerance, secondary obesity, or were under pharmacological treatment.

### Anthropometric Measures

Weight, height and waist circumference were measured according to standardized procedures [18]. All measurements were taken in underwear and without shoes. Weight was measured to the nearest 0.1 kg using a electronic column scale (SECA-780; Seca Ltd., Hamburg, Germany); height was measured to the nearest 0.1 cm using a stadiometer (SECA-220; Seca Ltd.), with the head in the Frankfort horizontal plane; waist circumference (WC) was measured with a non-elastic, flexible tape measured at the mid-point between the iliac crest and the lower edge of the ribs at the end of a normal expiration. Age was calculated in decimal units based on the date of the survey relative to birth date. BMI was calculated with measured height and weight and was standardised (z-BMI) by using age- and gender-normative data from the CDC [17,19]. Abdominal obesity was defined using the sex and age-specific 90^th ^waist circumference percentile by McCarthy *et al *[20]. Birth weight and length were obtained from the children health booklet.

### Clinical and Biochemical Measures

A physical examination was performed looking for characteristic abnormalities, specifically acanthosis nigricans. Puberty status was assessed according to pubic hair in boys and pubic hair and breast development in girls [21,22]. Blood pressure (BP) was obtained on the right arm with the patient seated, after rest, using a digital sphygmomanometer (OMRON M6) and appropriate sized cuff. After three measurements, the lowest blood pressure value was chosen. Children were classified according to sex, height and age-specific charts [23].

Baseline blood samples were collected in the morning (8:00 to 9:00) after an overnight fast (10 to 12 hours) by venipuncture, after a local application of a topical anesthetic patch (EMLA). Plasma and serum were separated by centrifugation. The glucose oxidase method (Siemens Healthcare Diagnostics Inc., Newark, DE, USA) was used to determine blood glucose levels. Serum lipids (total cholesterol (T-chol), HDL, LDL, triglycerides) were measured using an enzymatic colorimetric method (Siemens Healthcare Diagnostics Inc.). Very low density lipoprotein (VLDL) was calculated using the principles of the Friedewald formula, and the Apolipoprotein A-I and Apolipoprotein B were measured by an immuno-turbidimetric assay (Olympus America Inc., Center Valley, PA, USA). Plasma liver enzyme (alanine aminotransferase (ALT), aspartate aminotransferase (AST), gama-glutamyltransferase (GGT)) levels were measured using an enzymatic colorimetric method (Siemens Healthcare Diagnostics Inc.), and C-reactive protein (CRP) was determined by a turbidimetric immunoassay (Siemens Healthcare Diagnostics Inc.). Plasma and serum were frozen until further analysis of insulin, C-peptide, leptin, adiponectin and ghrelin at an external laboratory (ENDOCLAB, Porto). Insulin and C-peptide were measured by luminescence immunometric assay (Siemens Healthcare Diagnostics Inc.). Insulin resistance was determined by the Homeostasis Model Assessment of Insulin Resistance (HOMA-IR) calculated as the product of the fasting plasma insulin level (μUI/ml) and the fasting plasma glucose level (mmol/l), divided by 22.5 [24]. Leptin, adiponectin and ghrelin were quantified by RIA (Linco Research Inc., St. Charles, MO, USA).

### Definition of MS

MS was defined according to the National Cholesterol Education Program Adult Treatment Panel III criteria modified by Cook [10], with adjustment for fasting glucose according to the recent American Diabetes Association definition for impaired fasting glucose [25]. This definition was chosen since it is based in age- and gender-specific cutoffs and it has been used in several paediatric studies [8,26]. Thus, MS was considered if three or more of the following criteria were present: 1) abdominal obesity (WC ≥ 90^th ^percentile for age and sex) [20]; 2) fasting TG ≥ 110 mg/dl; 3) HDL ≤ 40 mg/dl; 4) systolic/diastolic BP ≥ 90^th ^percentile for age, sex and height [23]; 5) fasting glucose ≥ 100 mg/dl [25].

### Statistical Analysis

Descriptive statistics were executed by computing the mean and standard deviation (SD) for scale variables, or frequencies for nominal variables. Pearson and Spearman correlation coefficients were computed to evaluate the degree of association between variables. To study the independence between nominal categorical variables Chi-square test was used. Independent samples t test was performed in scale variables to evaluate the differences between two groups. Mann-Whitney test was used in ordinal variables to evaluate the differences between two groups. The normality of scale variables was assessed using the Kolmogorov-Smirnov test. Non parametric tests were applied to variables with a distribution significantly different from the Normal. We considered a significance level of 0.05. Statistical analysis was performed using the Statistical Package for the Social Sciences (SPSS version 14.0, 2005).

## Results

The anthropometric and clinical characteristics of the 82 children (48.8% boys and 51.2% girls) are shown in Table 1. A statistical significant difference between genders was found for height, z-IMC, WC and the presence of acanthosis, with boys presenting higher values. Mean plasmatic concentration of total cholesterol (169.4 ± 27.1 mg/dl), HDL (50.1 ± 9.7 mg/dl), LDL (106.9 ± 23.2 mg/dl), TG (77.7 ± 47.3 mg/dl), insulin (8.6 ± 4.2 μUI/ml), glucose (82.0 ± 6.7 mg/dl), AST (25.3 ± 4.9 U/L), ALT (20.3 ± 8.0 U/L) and GGT (15.6 ± 6.4 U/L) were within normal range for both genders. Plasma liver enzymes showed significant higher concentrations in boys: ALT (26.5 ± 5.5 vs 24.1 ± 4.0 U/L; p = 0.021), AST (22.8 ± 9.7 vs 17.8 ± 5.0 U/L; p = 0.005) and GGT (17.1 ± 8.1 vs 14.2 ± 3.6 U/L; p = 0.043). About three quarters of the sample were prepubertal (Tanner I: 74.4%; Tanner II: 25.6%). The prevalence of MS in our children was 15.8%, without significant differences between boys and girls. None of the children fulfilled the five criteria of the MS.

**Table 1:** Descriptive characteristics of the population study, according to gender.

	Boys (*n *= 40)	Girls (*n *= 42)	***p***
Age (y)	8.7 ± 0.8	8.6 ± 0.7	0.721^a^
Weight (kg)	43.5 ± 6.4	41.4 ± 6.0	0.128^a^
Height (cm)	136.8 ± 5.9	134.2 ± 5.6	0.049^a^
BMI (kg/m^2^)	23.14 ± 2.22	22.85 ± 2.07	0.530^a^
z-BMI	2.00 ± 0.29	1.86 ± 0.26	0.026^a^
Waist circumference (cm)	74.1 ± 5.8	71.5 ± 5.9	0.044^a^
Systolic BP (mmHg)	117.2 ± 8.1	115.4 ± 9.8	0.402^a^
Diastolic BP (mmHg)	64.0 ± 10.9	61.9 ± 8.3	0.330^a^
HOMA-IR	1.71 ± 0.90	1.79 ± 0.89	0.798^b^
Birth weight (g)	3523 ± 429	3337 ± 472	0.072^a^
Acanthosis (*n*; %)	13 (32.5%)	5 (11.9%)	0.024^c^
Metabolic Syndrome (*n*; %)	6 (15.0%)	7 (16.7%)	0.836^c^

Data presented as mean ± SD for continuous variables and as frequency (%) for dichotomous variables.

^a ^t-student test for the differences between genders.

^b ^Mann-Whitney for the differences between genders.

^c ^Chi-square test for the independence between gender and each variable.

Between the assessment at school and the first assessment at the Hospital, 14 (17.1%) children who were classified initially as obese (≥ 95^th ^BMI percentile) became overweight (85-95^th ^BMI percentile). In addition to the significantly higher values in anthropometric parameters, the levels of systolic BP (117.2 ± 9.1 vs. 111.9 ± 7.4 mmHg; p = 0.047) and C-peptide (1.0 ± 0.5 vs. 0.8 ± 0.3 ng/ml; p = 0.010) were also found significantly higher in obese children. Glucose, insulin and HOMA-IR showed a trend towards higher levels in obese subjects, as well as blood lipids (except HDL), leptin and GGT, without statistical significance (data not shown). None of the overweight children had 3 or more diagnostic criteria for MS or presented acanthosis. In obese, 13 (19.1%) presented MS and 18 (26.5%) presented acanthosis.

As shown in Table 2, BMI, z-BMI, WC, systolic BP and the presence of acanthosis were significantly higher in MS children than in non-MS sufferers. The number of components of MS was higher in children with higher z-BMI (ρ = 0.411, p < 0.001). We found significant differences between the presence and the absence of MS for several biochemical characteristics (Table 3). The number of criteria for MS correlated with several characteristics of the population, however most of these correlations were weak (|ρ| < 0.5), except for systolic BP (ρ = 0.682, p < 0.001).

**Table 2:** Descriptive characteristics of the population study according to the presence or absence of MS.

	Without MS (*n *= 69)	With MS (*n *= 13)	***P***
Age (y)	8.7 ± 0.7	8.4 ± 0.8	0.167^a^
Weight (kg)	41.9 ± 6.3	45.1 ± 5.2	0.091^a^
Height (cm)	135.4 ± 5.9	136.2 ± 5.5	0.635^a^
BMI (kg/m^2^)	22.75 ± 2.13	24.23 ± 1.74	0.021^a^
z-BMI	1.89 ± 0.26	2.15 ± 0.25	0.002^a^
Waist circumference (cm)	72.1 ± 5.9	76.4 ± 5.0	0.016^a^
Systolic BP (mmHg)	114.9 ± 8.3	123.5 ± 9.2	0.001^a^
Diastolic BP (mmHg)	62.7 ± 10.2	63.9 ± 6.7	0.675^a^
Birth weight (g)	3443 ± 442	3328 ± 546	0.412^a^
Gender (M/F)	34/35	6/7	0.836^b^
Tanner (I/II)	49/20	12/1	0.107^b^
Acanthosis (*n*; %)	11 (15.9%)	7 (53.8%)	0.002^b^

Data presented as mean ± SD for continuous variables and as frequency for dichotomous variables.

^a ^t-student test for the differences between absence or presence of MS.

^b ^Chi-square test for the independence between absence or presence of MS and each variable.

**Table 3:** Biochemical characteristics of the population study according to the presence or absence of MS.

	Without MS (*n *= 69)	With MS (*n *= 13)	***p***
Glucose (mg/dl)	81.8 ± 6.6	82.9 ± 7.4	0.580^a^
Insulin (μUI/ml)	8.1 ± 3.6	11.3 ± 6.3	0.037^b^
C-Peptide (ng/ml)	0.9 ± 0.5	1.2 ± 0.5	0.044^b^
HOMA-IR	1.64 ± 0.77	2.31 ± 1.25	0.028^b^
T-chol (mg/dl)	167.6 ± 25.5	179.2 ± 33.9	0.156^a^
HDL (mg/dl)	51.9 ± 8.9	40.4 ± 8.4	< 0.001^a^
LDL (mg/dl)	104.5 ± 21.5	119.7 ± 28.3	0.030^a^
Triglycerides (mg/dl)	63.4 ± 19.2	153.7 ± 74.7	< 0.001^b^
VLDL (mg/dl)	12.7 ± 3.8	30.7 ± 14.9	< 0.001^b^
T-chol/HDL	3.27 ± 0.50	4.54 ± 0.98	< 0.001^b^
Apolipoprotein A-I (mg/dl)	126.2 ± 14.9	114.1 ± 14.7	0.008^a^
Apolipoprotein B (mg/dl)	75.4 ± 13.8	87.7 ± 18.2	0.006^a^
Apolipoprotein A-I/B	1.73 ± 0.37	1.36 ± 0.37	0.001^a^
C-reactive protein (mg/dl)	0.38 ± 0.81	0.31 ± 0.23	0.283^b^
Leptin (ng/ml)	10.6 ± 5.8	15.5 ± 7.8	0.011^a^
Adiponectin (ng/ml)	26428.1 ± 12915.0	26136.9 ± 11291.1	0.940^a^
Ghrelin (pg/ml)	1051.1 ± 420.3	985.1 ± 380.7	0.600^a^
AST (U/L)	25.0 ± 6.3	26.8 ± 6.3	0.437^b^
ALT (U/L)	19.4 ± 7.2	25.2 ± 10.3	0.011^b^
GGT (U/L)	14.7 ± 4.1	20.6 ± 12.0	0.008^b^

Data presented as mean ± SD.

^a ^t-student test for the differences between absence or presence of MS.

^b ^Mann-Whitney for the differences between absence or presence of MS.

**Figure 1:**
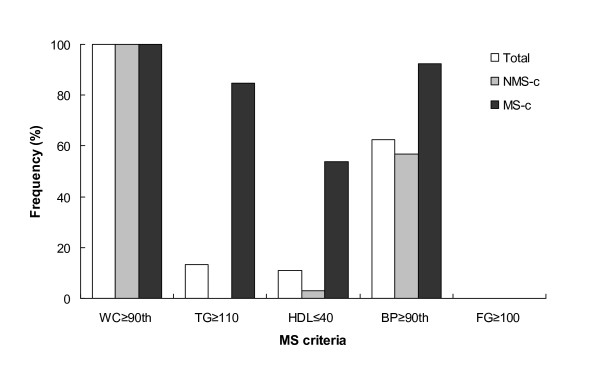
**Frequency of the components of MS in total children, children without MS and children with MS**. MS: Metabolic syndrome; NMS-c: children without MS (*n *= 69); MS-c: children with MS (*n *= 13); WC: waist circumference; TG: triglycerides; HDL: HDL-cholesterol; BP: blood pressure; FG: fasting glucose.

All children presented abdominal obesity (WC ≥ 90^th^), while high BP (≥ 90^th^) was the second most frequent component of MS (62.6%) (Figure 1). Frequency of high TG and low HDL was 13.4% and 11.0%, respectively. Abnormal fasting glucose values were not identified in any of the children, however IR occurred in 8.5% (23.1% in MS-children vs. 5.8% non-MS), hyperinsulinemia (plasma insulin >15 μUI/ml) in 7.3% and acanthosis was present in 22.0%.

Leptin correlated positively (p < 0.05) with BMI, z-BMI, WC, insulin, HOMA-IR, C-peptide, T-chol, LDL, TG, Apolipoprotein B, ALT, GGT and CRP, and negatively with HDL and Apolipoprotein A-I/B, but most correlations were weak (|R|,|ρ|<0.5). Adiponectin showed a negative (p < 0.05) but weak (|R|, |ρ|<0.5) correlation with diastolic BP and birth weight, without any significant correlation with obesity. Also a negative (p < 0.05) and weak (|R|, |ρ|<0.5) correlation was found between ghrelin and age, weight, BMI, systolic BP, insulin and HOMA-IR.

## Discussion

Our study presents the first Portuguese report on the frequency of MS and associated metabolic complications among overweight and obese children aged 7-9 years. Since our study was population-based, not hospital-based, the severity of obesity was moderate (z-BMI mean: 1.93 ± 0.28). We found a high prevalence of MS (19.1%) in obese children, while it was absent in the overweight. Nevertheless, all children presented abdominal obesity, a criterion of MS. The observed prevalence of MS in our study is similar to data from other European countries [8,15,26]. However, when considering the definition applied, large differences are noticed [8,15]. Thus, a consensual paediatric definition of MS is needed in order to better compare studies and populations. Boys and pubertal subjects usually present higher frequencies of MS [9,10], however we did not found a significant difference in the prevalence of MS according to gender or pubertal status.

Childhood obesity is frequently associated with cardiovascular and metabolic disturbances [13]. In the present study, several biochemical parameters failed to present a significant difference between overweight and obese children, except for systolic BP and C-peptide levels that were significantly higher in obese. This lack of difference may be due to the moderate grade of obesity and lower SD of z-BMI of our sample. We observed that children with MS presented higher z-BMI and largest significant differences in clinical and biochemical characteristics compared to those without MS. The reason why some obese children developed this syndrome, while others do not is still unknown [16]. This may be due to the presence of other underlying factors, such as IR. Indeed, IR seems to be an important pathophysiologic event contributing to MS, becoming more important than overall adiposity in the development of this syndrome [5,27]. In fact, in our study IR was lower in the obese relatively to MS-children. This alerts to the possibility that obese children with 2 or more MS components might form a high risk group within the obese population.

All evaluated children presented abdominal obesity, which is related with increased metabolic risk factors for DM2 and CVD [28]. Elevated BP was the second most common feature of MS, reaching about 2/3 of our sample, which is a remarkably high frequency. In obese children from several European countries, a lower frequency (38%) was reported [15], similar to a study with US children (7-9 years-old) [16]. The differences found may be do to different methodology, since we used a digital sphygmomanometer which may overestimate blood pressure levels. It also may be due to distinct dietary habits in our population, namely sodium intake. Systolic BP was significantly (ρ = 0.682; p < 0.001) correlated to the number of factors of MS.

Dyslipidemia is less prevalent, however the lipid profile can be considered more 'atherogenic' in MS children, with higher TG, LDL, VLDL and apolipoprotein B, and lower HDL and apolipoprotein A-I. Indeed, it is well established that atherosclerosis begins in childhood and is associated with several risk factors for MS [26].

Although the younger age of our sample, the children with MS already presented acanthosis, elevated insulin and C-peptide levels and increased HOMA-IR. Despite this, fasting glucose levels were normal which reflects the range of abnormalities of glucose homeostasis associated with childhood obesity. In fact, even in the obese, high fasting glycemia is not common in the paediatric age [29]. This suggests that impaired glucose levels or pre-diabetes develops later than the other MS components, while IR is the earlier and predominant abnormality of glucose metabolism found in obesity [30]. Indeed, excess weight in children and adolescents may serve to accelerate the onset of DM2 in childhood [5].

Higher levels of hepatic enzymes (ALT, AST and GGT) were found in boys and in children with MS and obesity, although within normal range values. This gender-difference may be due to a larger liver mass in males [31]. Non-alcoholic fatty liver disease is considered the hepatic manifestation of the MS in adults [32]. Currently, this condition is related with child obesity and considered a major cause of abnormal liver function tests in paediatric populations [31,32].

In our sample, leptin levels were correlated to obesity, glucose metabolism, an 'atherogenic' lipid profile and to liver function. Consequently, the presence of MS was associated with significant higher levels of this adipose-tissue hormone. Leptin is described to be positively correlated with both BMI and fasting insulin levels [33]. Chronic insulin increase favours an increase in leptin, which in turn would perpetuate the increase in insulin and favour the onset of IR. This leptin-insulin association begins before puberty and may be connected to the onset of MS [33].

Bacha et al [34] reported that adiponectin is inversely associated with obesity and is an important determinant of insulin sensivity and HDL in children. However, we did not verify these relations. This is possibly due to the small sample size and more homogeneous characteristics of our sample, without severe obese children. In this study, adiponectin levels showed a weak and negative correlation with diastolic BP and birth weight.

Ghrelin, an orexigenic peptide secreted by the stomach, is documented to be inversely correlated to obesity, insulin and IR indexes in children [35,36], such as confirmed in our study. The negative relationship between fasting ghrelin concentration and obesity might be explained by an inhibitory effect of insulin on ghrelin, since a higher IR is associated with visceral fat accumulation [35].

## Conclusions

Our results showed a significant prevalence of MS and its cardiometabolic complications in Portuguese obese schoolchildren. Abdominal obesity was present in all children and the frequency of elevated BP levels was markedly high. None of the children presented impaired fasting glucose. The fact that the clustering of metabolic risk factors in childhood predicts the development of MS into adulthood highlights the importance of better understanding the longer-term health implications of this syndrome in children. Also, a consensual definition of paediatric MS is needed for proper screening, evaluation and treatment of children at risk or with MS. The implementation of preventive measures is urgently required since lifestyle modifications and weight loss may lead to favourable changes in risk factors.

## List of abbreviations

ALT: Alanine aminotransferase; AST: Aspartate aminotransferase; BMI: Body Mass Index; BP: Blood Pressure; CDC: Centers of Disease Control; CRP: C-reactive protein; CVD: Cardiovascular diseases; DM2: Type 2 Diabetes Mellitus; GGT: Gama-glutamyltransferase; HDL: High density lipoprotein; HOMA-IR: Homeostasis model assessment of insulin resistance; IR: Insulin resistance; LDL: Low density lipoprotein; MS: Metabolic syndrome; SD: standard deviation; T-chol: Total cholesterol; TG: Triglycerides; VLDL: Very low density lipoprotein; WC: Waist circumference; z-BMI: standard deviation score of Body Mass Index.

## Competing interests

The authors declare that they have no competing interests.

## Authors' contributions

CP developed the study protocol, participated in children assessment, data analysis and writing the manuscript. BMPMO completed the statistical analysis and provided advice regarding interpretation and presentation of results. IA participated in children assessment and contributed to the revising of the manuscript. CSP and MDVA provided advice on study design and contributed to the writing of the manuscript. FC participated in protocol development, data analysis and contributed to the writing of the manuscript. All authors read and approved the final manuscript.
